# Effect of fabrication process on contact resistance and channel in graphene field effect transistors

**DOI:** 10.1038/s41598-024-58360-9

**Published:** 2024-04-22

**Authors:** Babak Khosravi Rad, Amir Hossein Mehrfar, Zahra Sadeghi Neisiani, Mahdi Khaje, Abdollah Eslami Majd

**Affiliations:** 1 Optoelectronics and Nanophotonics Research Group, Faculty of Electrical and Computer Engineering, Tarbiat Modares University, Tehran, Iran; 2 Faculty of Electrical and Computer Engineering, Malek Ashtar University of Technology, Tehran, Iran

**Keywords:** Applied physics, Electronics, photonics and device physics

## Abstract

Contact resistance, as one of the main parameters that limits the performance of graphene-based transistors, is highly dependent on the metal-graphene contact fabrication processes. These processes are investigated and the corresponding resistances are measured based on the transfer length method (TLM). In fabrication processes, when annealing is done on chemical vapor deposition (CVD)-grown graphene samples that are transferred onto SiO_2_/Si substrates, the adhesion of graphene to the substrate is improved, and poly methyl methacrylate (PMMA) residues are also reduced. When the metal deposition layer is first applied to the graphene, and then, the photolithography process is performed to define the electrodes and graphene sheet, the graphene-metal contact resistance is better than that in other methods due to the removal of photoresist residues. In fact, by changing the sequence of the fabrication process steps, the direct contact between photoresist and graphene surface can be prevented. Thus, the contact resistance is reduced and conductivity increases, and in this way, the performance of graphene transistor improves. The results show that the fabrication process has a noticeable effect on the transistor properties such as contact resistance, channel sheet resistance, and conductivity.‌ Here, by using the annealing process and changing the order of photolithography processes, a contact resistance of 470 Ω μm is obtained for Ni-graphene contact, which is relatively favorable.

## Introduction

Unique features of graphene, such as no forbidden gap, high carrier mobility^1,2^, high saturation speed^3^, and thermal stability^4,5^, have made it an excellent candidate for different optical and electronic applications^6,7^. A critical application of graphene is in the fabrication of high-frequency graphene transistors and photodetectors^8–13^. The performance of these transistors is influenced by the inherent mobility of carriers in the channel and the metal-graphene contact resistance^8^. The effect of carrier mobility in devices with longer channel length is more considerable than the effect of contact resistance. However, when the channel length is reduced, the role of contact resistance becomes more critical and dominant in improving the device speed^8^. The contact resistance under the mentioned conditions limits the performance of short-channel graphene transistors and prevents them from reaching higher frequencies^14–16^. Moreover, high contact resistance limits the responsivity of graphene photodetectors^11–13^.

The metal-graphene contact resistance depends on some factors, such as the metal type^17,18^, contact interface contamination due to photoresist residues^19^, moisture, and trapped charges at the contact interface^20^. Moreover, the metal deposition technique can affect contact resistance^21^. The metals that are used in metal-graphene contacts are Cr, Ti, Cu, Au, Ni, Pd, Pt, and Co^17,22–24^. Although Pd and Au have a low contact resistance in contact with graphene, they exhibit poor adhesion to the substrate^25^. Metals Cr and Ti are characterized by good adhesion and desirable contact resistance in contact with Au and Pd^26–28^. In this article, the mentioned metals are not used as contact electrodes. This is because the etchant used for these metals in the two-in-one process^29^ degrades graphene, but Ni can be used as the electrode in graphene field effect transistors (GFETs) due to its strong adherence to graphene^30^, compatibility with CMOS technology^31^, and lower contact resistance compared to other metals^22,32^.

Metal-graphene contact resistance can be characterized by some methods, such as two-point probes, four-point probes, and transfer length method (TLM). The two-point probe method is a simple and basic method of measuring contact resistance. This method is not appropriate for small contact areas^33^. The four-point probe method is also appropriate for measuring the sheet resistance of surface layers and specific bulk resistivity of materials^34^, so it is used for measuring the metal-graphene contact resistance^35,36^. Nevertheless, TLM is the most common method for characterizing the contact and sheet resistance of the graphene channel since this method has a simpler fabrication process and presents more comprehensive data^31,37–42^.

The works conducted in recent years are summarized as follows: In 2016, Carlos Alvardo Chavarin et al. showed that due to the presence of 3–4 nm photoresist residues at the metal-graphene contact interface, the contact resistance for the top-contact mode is higher than 4 kΩ μm^19^. In 2017, Mehrdad Shaygan et al. showed that the contact resistance for the top-contact mode is of the order of several kΩ μm due to the presence of photoresist residues; moreover, they showed that the value of contact resistance for edge-contacted Ni/Al is equal to 2.5 ± 1 kΩ μm^31^. In 2019, Liu Fengyuan et al. reported a contact resistance of less than 200 Ω μm for a bottom-contact electrode, and they obtained a meager value of 65 Ω μm for contact resistance with electron beam lithography^43^. Also, it has been shown that the annealing process improves contact resistance^44–46^. In addition, reducing the contact resistance improves the cut-off frequency and gm of FET^47–50^. In the present study, photolithography is used to investigate three techniques of implementing TLM measurements. Moreover, this study uses the photolithography process to implement the metal-graphene contact for transfer length measurement. In other words, three metal-graphene contact models are used and the contact characteristics are investigated using TLM. Also, for each of the metal-graphene contact processes, the current of the graphene channel is obtained as a function of the gate voltage and drain-source voltage, and the conductivity is obtained as a function of gate voltage.

## Fabrication process

The TLM measurement was used to characterize the metal-graphene contact resistance. The TLM can be implemented using three techniques including graphene on top of metal, two-in-one, and hybrid techniques, which are used to fabricate metal-graphene contacts.

In all methods, to perform the photolithography process, photoresist was used at 3000 rpm for 45 s and annealing was performed at 115 °C for 90 s. Accordingly, electrodes and graphene sheets were created on the samples. Also, 30 nm of nickel metal was deposited at a rate of 1 A/s using the electron beam evaporation method under a pressure of 2 × 10^−6^ Torr. A 0.35 mol/L FeCl_3_ solution was used for etching nickel, and the samples were dipped in acetone for 30 min to remove residues. The graphene, which was used in this study, was purchased from Graphena and grown by the chemical vapor deposition method. The characteristics of the graphene were a uniformity of 80 nm, a mobility of 850 $\frac{{{\text{cm}}^{2} }}{{{\text{v}}\,{\text{s}}}}$, and grain boundaries ranging from 20 to 30 μm. Then, chemical vapor deposition (CVD) graphene samples with a poly methyl methacrylate (PMMA) support layer were transferred to the SiO_2_/Si substrates in the laboratory, and graphene etching was performed using oxygen plasma under a pressure of 300 mTorr and a power of 50 W within 5 min.

**Figure 1:**
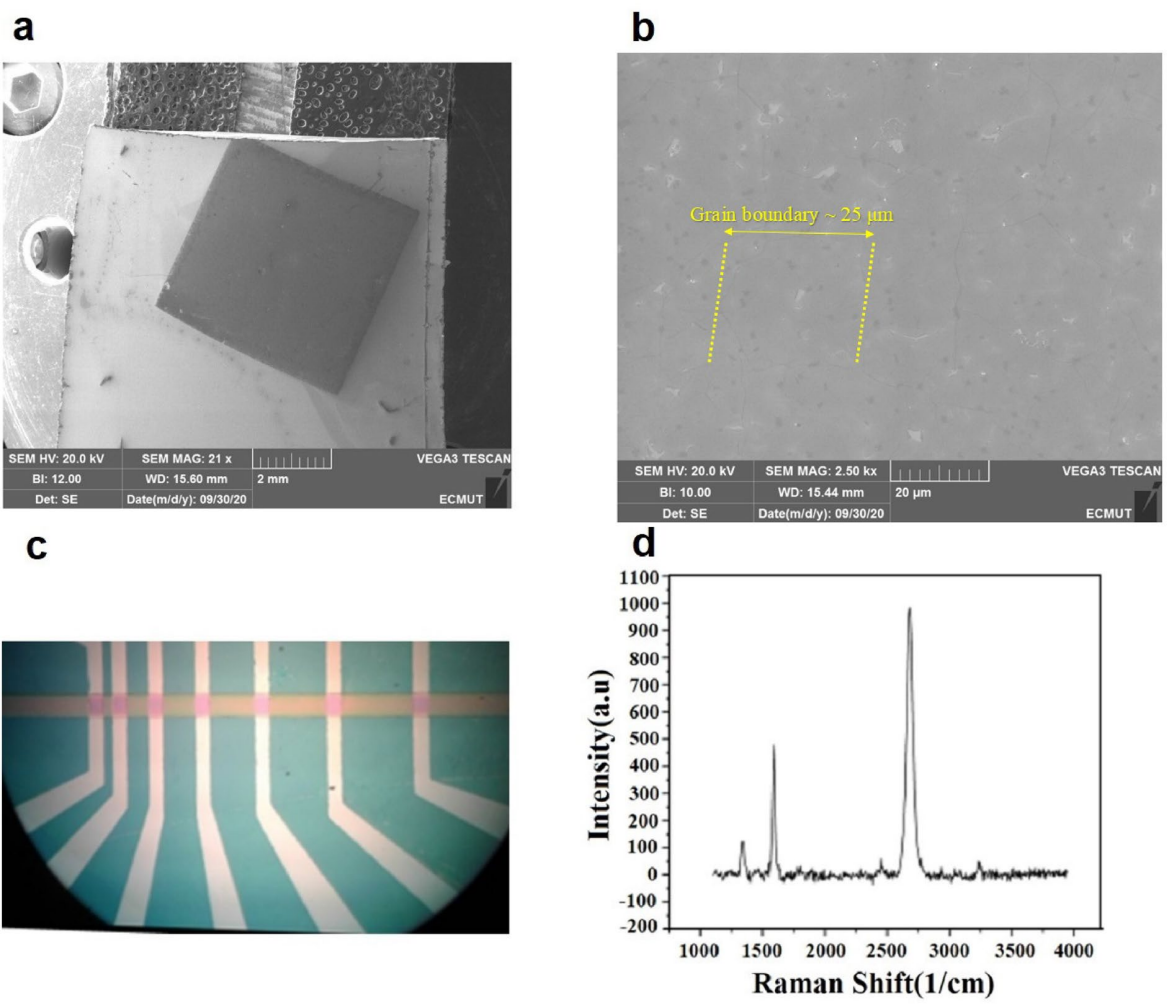
(**a**) SEM image of graphene on SiO_2_/Si at 21× magnification, (**b**) SEM image of graphene on SiO_2_ at 2500× magnification, (**c**) optical microscope image of patterning the Nickel layer, and (**d**) Raman spectroscopy image of graphene on SiO_2_.

In Fig. 1, a general view of the transferred graphene and the TLM structure are shown. Figure 1a,b show the scanning electron microscopy (SEM) images of the graphene on the SiO_2_/Si substrate, which were taken using a TESCAN VEGA3 device before the photolithography process. The graphene boundaries that show the residues were removed, as shown in Fig. 1c at 2500× magnification. The optical microscope image of the sample after TLM implementation and the Raman spectroscopy image of graphene on SiO_2_/Si are shown in Fig. 1c,d, respectively.

### Graphene on top of metal

**Figure 2:**
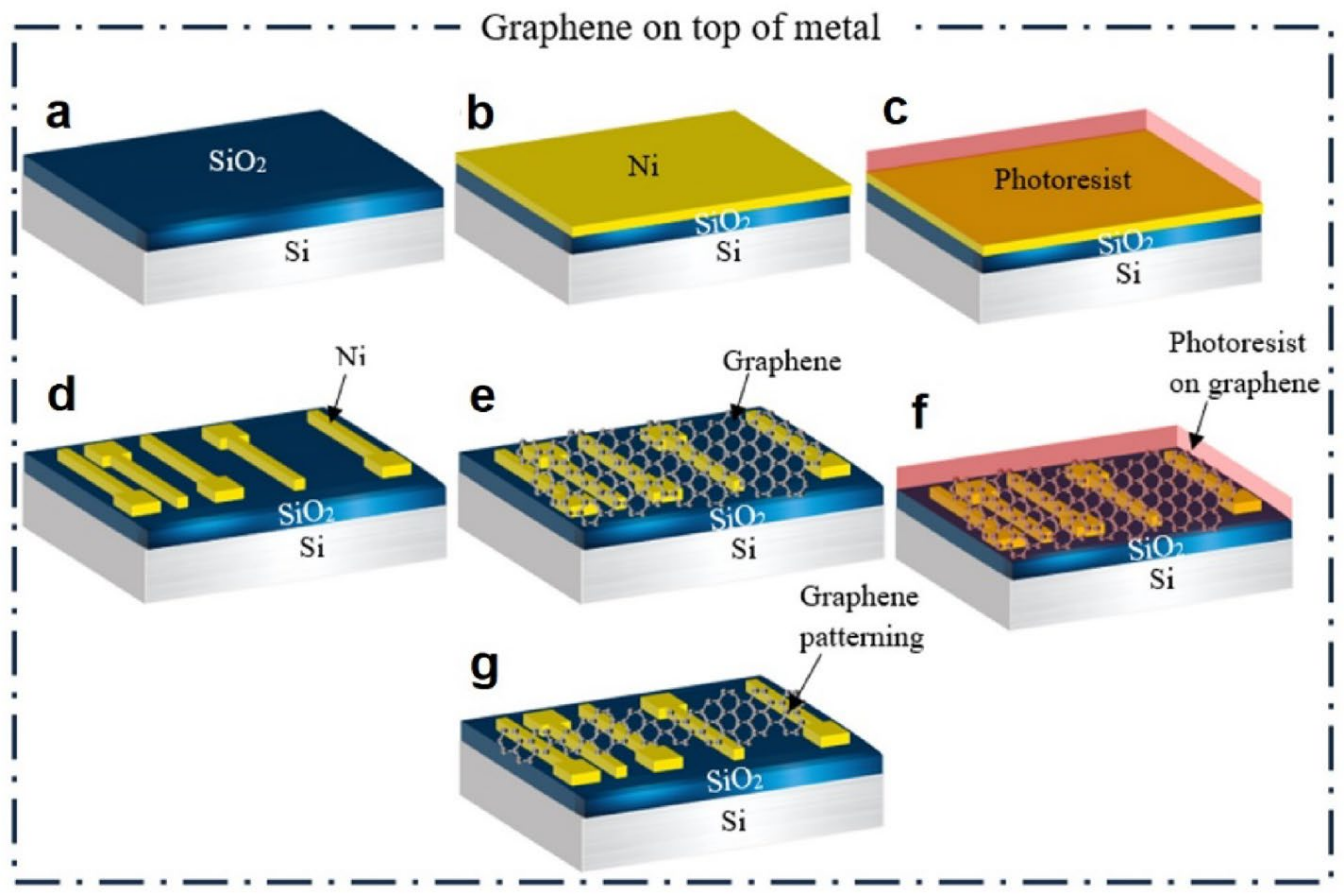
The fabrication process in the graphene on top of metal technique.

To implement the technique of graphene on top of metal, Ni was first deposited on SiO_2_/Si substrate using an electron beam evaporation technique, as shown in Fig. 2b. Then, using photolithography process, the TLM layout was patterned on the sample, and metal electrodes were made by etching Ni using FeCl_3_ solution (0.35 mol/lit), as shown in Fig. 2d. Next, an acetone solution was used to clean the photoresist on the electrodes, and the graphene samples grown by CVD were transferred to the electrodes (see Fig. 2e) and patterning was performed on them, as shown in Fig. 2f,g. The steps for this process are shown in Fig. 2.

### Hybrid method

**Figure 3:**
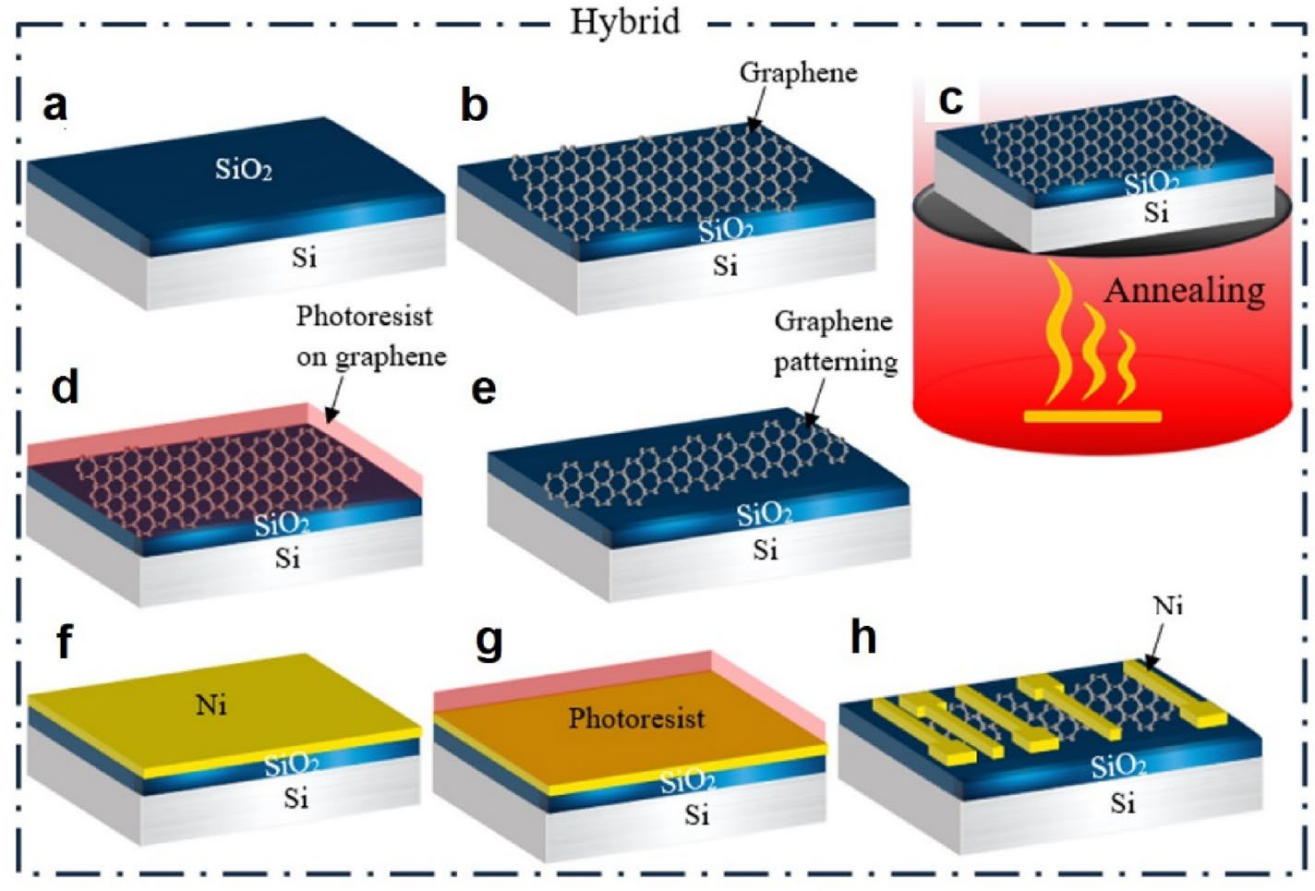
The fabrication process in the hybrid method.

The graphene was transferred to the Si/SiO_2_ substrate, as shown in Fig. 3b. Then, the graphene was annealed at 550 °C under a pressure of 4 × 10^−6^ Torr for 3 h (see Fig. 3c). Vacuum annealing was used to clean the PMMA residues. Next, the graphene was patterned by photolithography and etched by oxygen plasma, as shown in Fig. 3d,e. The sample was immersed in acetone for 5 min and then placed in N-Methyl-2-pyrrolidone (NMP) solution at 75 °C for 15 min to clean the photoresist and other residues. After cleaning the graphene sheet, nickel was deposited, as shown in Fig. 3f. The photolithography process was repeated, and the graphene sheet was patterned, as shown in Fig. 3h. Unlike the two-in-one process in which the graphene sheet is made in the last step, in this method, the graphene sheet is made in the first step. The fabrication process in the hybrid method is shown in Fig. 3.

### Two-in-one process

**Figure 4:**
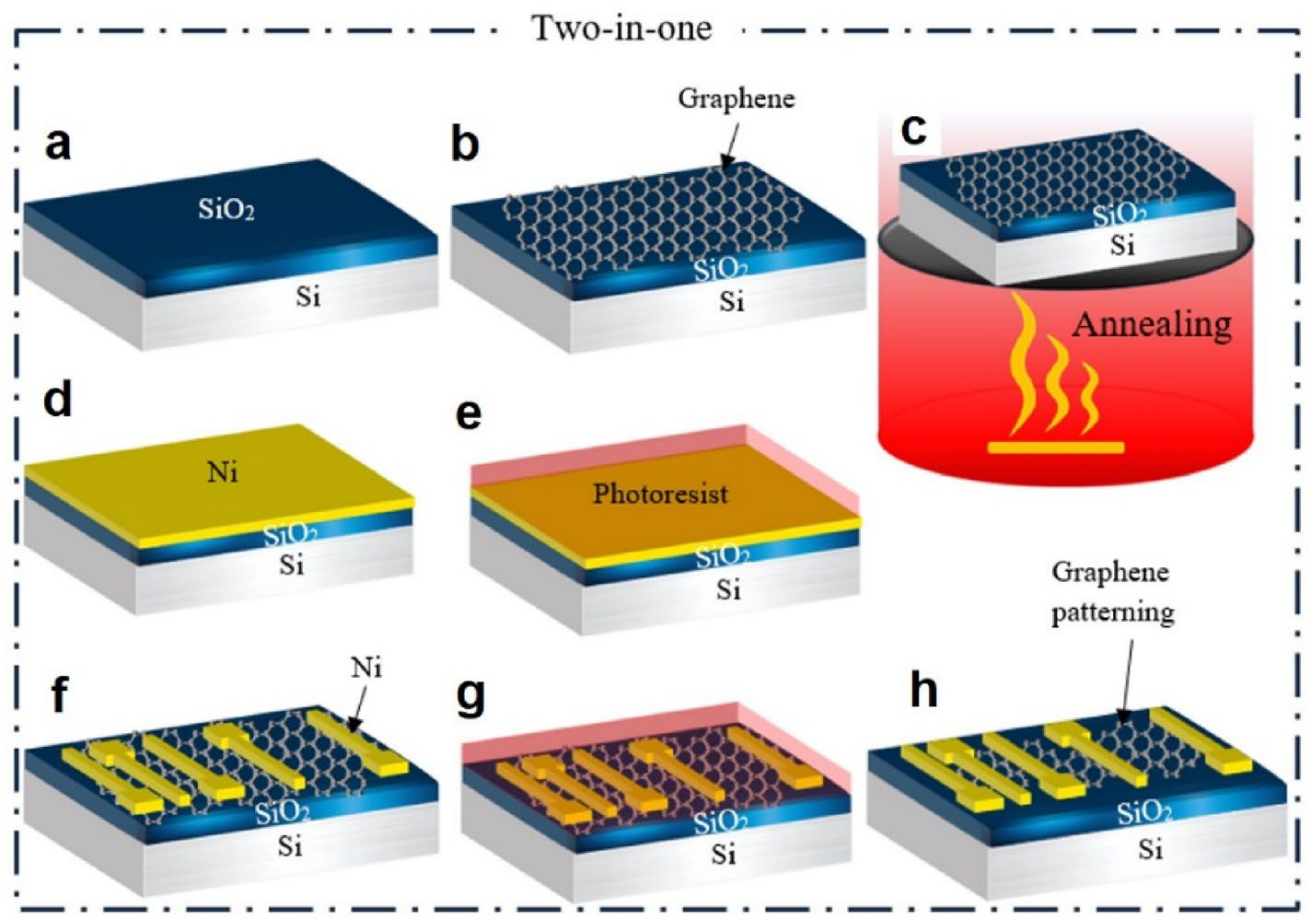
The fabrication process in the two-in-one technique.

Similar to the hybrid method, first, the graphene was transferred and annealed, as shown in Fig. 4a–c. Then, Ni was deposited after transferring graphene onto a SiO_2_/Si substrate (see Fig. 4e). Using photolithography followed by etching Ni, electrodes were made on graphene, as shown in Fig. 4e,f. In Fig. 4g,h, photolithography was performed once again to define the graphene sheet considering that photoresist and PMMA residues are important factors in increasing contact resistance. Therefore, by changing the sequence of the photolithography and metal deposition processes, these undesirable factors can be removed in this method. The fabrication process in the two-in-one process is shown in Fig. 4.

In two-in-one process, the metal not only acts as electrode but also serves as an interface layer between the graphene and photoresist, preventing the contamination of the metal-graphene contact interface when forming contacts. Moreover, after the mentioned process and before fabrication, the graphene samples were annealed according to the conditions mentioned in the hybrid method. This was done to improve the adhesion of graphene to the substrate and to eliminate the PMMA residues resulting from the transfer process. The transferred graphene is covered by a protective layer, i.e. PMMA. This layer is an organic material whose atomic chain breaks under the influence of temperature, causing it to escape from the surface in the form of a gas. Thus, this disturbing barrier disappears and improves the contact resistance between the metal and graphene as well as improves the adhesion to the substrate because the bonding of graphene carbon atoms with metal and substrate is facilitated.

## Results and discussion

**Figure 5:**
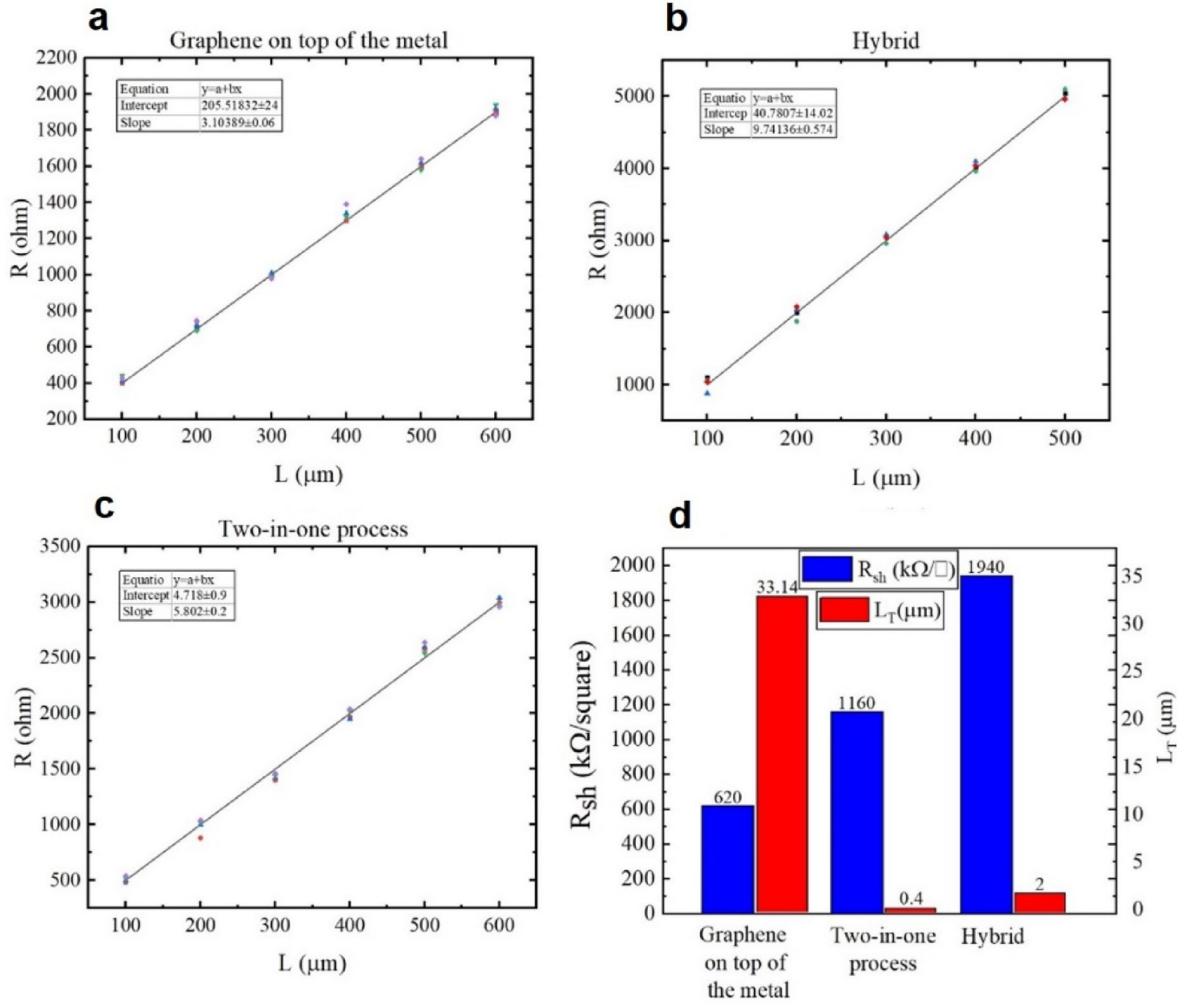
TLM measurement of samples; (**a**) graphene on top of metal, (**b**) hybrid method, (**c**) two-in-one process, and (**d**) comparison of sheet resistance and L_T_ in these three methods.

In the TLM method, the IV measurements were performed using an HP 4145B Semiconductor Parameter Analyzer. In the graphene on top of metal technique, the obtained contact resistance is 20.5 kΩ μm, which is relatively large (see Fig. 5a). This large value is due to the photoresist residues between the electrodes and graphene, the oxidation of Ni surface, and the formation of an interface layer between the metal and graphene.

In the hybrid method, after implementing the TLM method, measurements were made. The corresponding diagram is shown in Fig. 5b. In this case, the contact resistance is 4 kΩ μm, which is a relatively good value compared to the method of graphene on top of the metal. In this method, NMP is a cleaning solution that is essential in removing photoresist residues and PMMA. This is also confirmed by the obtained low contact resistance value. In addition, no metal oxide layer is formed at the graphene-Ni contact, and the interface is clean.

Photoresist residues and PMMA are the most critical factors in increasing contact resistance. Therefore, to eliminate these factors and improve the adhesion of graphene to the substrate, the two-in-one process was used for metal-graphene contact, i.e., for implementing TLM measurement. Figure 5c shows the TLM diagram. According to this figure, a contact resistance of 470 Ω μm, which is a good value for a metal-graphene contact, was obtained. A lower contact resistance is obtained when the metal is directly deposited on the graphene, and the graphene sheet is formed. This is because the graphene sheet has no contact with the photoresist as there are no photoresist residues between the metal and graphene (note that photoresist residue is the most undesirable factor contributing to the contact resistance). Figure 5d shows the sheet resistance, which is calculated as ${R}_{sh}=m\times w$, where $m$ is the gradient of the line and $w$ is the width of the channel. The sheet resistance is different in each of the three discussed methods. Each method has its own unique fabrication process, and the fabrication process affects graphene sheet resistance. In the three methods, the sheet resistances are acceptable. As shown in Fig. 5d, the hybrid and two-in-one processes have a low L_T_, so the effective area is improved because it is directly related to L_T_.

In this method, the contact metal acts as the electrode and photoresist-graphene interface. Accordingly, the contact resistance in this method is much lower compared to that obtained in other methods. Table 1 presents the measurement results of all three types of metal-graphene contact.

**Table 1:** Results of TLM measurement on graphene for different types of metal-graphene contact.

Measurement method	Graphene on top of metal	Hybrid	Two-in-one process
Contact resistance (kΩ.μm)	20.5	4	0.47
Sheet resistance (Ω/square)	620	1940	1160
Transfer length (μm)	33.14	2	0.4

**Figure 6:**
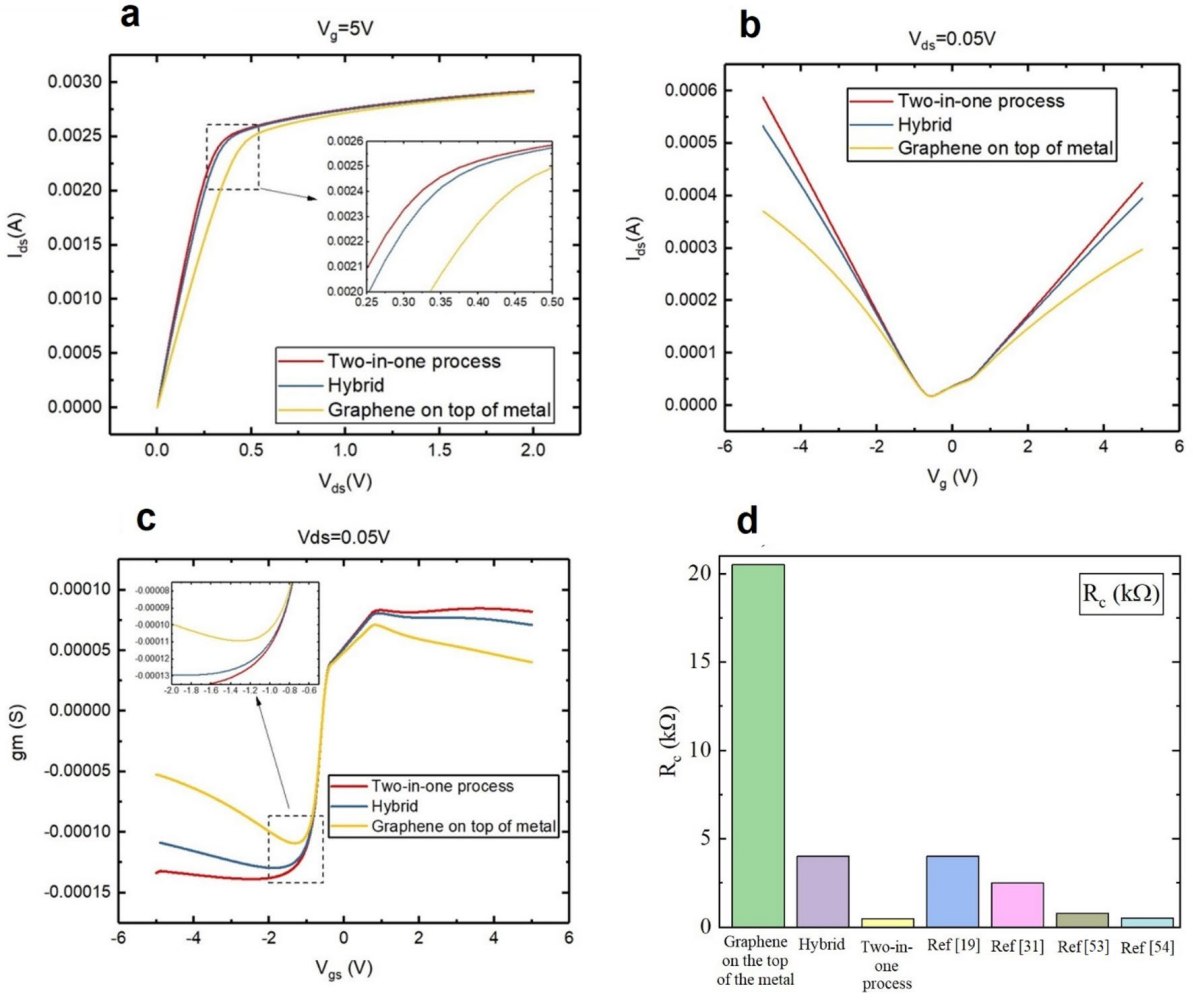
(**a**) graphene channel current as a function of drain-source voltages at V_gs_ = 5 V in different structures, (**b**) graphene channel current as a function of gate voltages at V_ds_ = 0.05 V in different structures, (**c**) g_m_ as a function of gate voltages at V_ds_ = 0.05 V in different structures, and (**d**) comparison of the three methods with previous approaches.

The contact resistances from the experimental results were used in Lumerical simulation software. We simulated a GFET with the back-gate voltage. The experimental contact resistances were used as simulation values in the software. This software assumes a parabolic band dispersion to solve a material-like graphene. According to this assumption, the graphene is considered a semiconductor with the following physical values: a thickness of 0.75 nm, a band gap of about 0.2 eV, and an effective mass (${m}^{*}$) of about 0.4614. We simulated graphene as a semiconductor with a very small bandgap. The graphene channel current was simulated as a function of different drain-source voltages and gate voltages for two-in-one, hybrid, and graphene on top of metal processes. In Fig. 6a, the graphene channel current is plotted as a function of drain and source voltages at a constant gate voltage of 5 V for different structures. The two-in-one process has a lower contact resistance than the hybrid and graphene on top of metal processes. Additionally, the transfer curve moves faster from the linear region to the saturated region. In Fig. 6b, the graphene channel current is plotted as a function of gate voltages at constant drain-source voltages of 0.05 V for different structures. The contact resistance is increased in GFET^51^, and transfer characteristics are affected by the changes in resistance^32^, which is evident in Fig. 6b. Using the data of Fig. 6b and equation $gm=\frac{\partial Ids}{\partial Vgs}$, the conductivity (g_m_(S)) was plotted as a function of gate voltages for different structures (see Fig. 6c).

Figure 6d compares the three methods presented in this study with the previous approaches. The two-in-one process has the lowest contact resistance compared to other structures.

The contact resistance is very effective on graphene-based devices. For example, in the graphene field effect transistor, when the contact resistance is reduced, f_T_ and f_max_ increase. In a graphene photodetector, which uses the photovoltaic effect, when the contact resistance is reduced, bandwidth increases. In a photogating photodetector based on graphene, the gain can be related to the change in conductivity Δ$\sigma$ = Δn × e × µ due to the light-induced modification of the graphene carrier concentration^52^.

## Conclusion

In this study, it was shown that the adhesion of graphene to the substrate is improved by annealing under a pressure of 4 × 10^−6^ Torr and at a temperature of 550 °C for 3 h. Different models of metal-graphene contact were investigated, and the graphene channel current was simulated as a function of gate voltages and drain-source voltages of these models. It was shown that the contact resistance at the metal-graphene contact is large when graphene is placed on nickel electrode. This can be attributed to the thin oxide layer at the metal-graphene interface, weak adhesion, and the presence of photoresist residues. However, in the hybrid method, the mentioned issues are solved to a large extent due to using NMP solution, which more effectively cleans the graphene surface and the metal-graphene interface, so the contact resistance becomes smaller. Moreover, the graphene sheet is exposed to the photoresist, which is the main reason behind the large contact resistance values. In the two-in-one process, the main factor causing considerable metal-graphene contact resistance was eliminated by depositing the metal right after transferring the graphene and preventing direct contact between the photoresist and graphene. In this method, using photolithography, a contact resistance of 470 Ω μm was obtained, which is a suitable value because the lowest contact resistance reported in most of the literature is greater than 4 kΩ μm. The obtained experimental data were used in the simulation model, and the effects of contact resistance on Dirac curves and conductance were simulated.

## Data Availability

All data is presented in this article.
